# Delays in Patient Presentation and Diagnosis for Buruli Ulcer (*Mycobacterium ulcerans* Infection) in Victoria, Australia, 2011–2017

**DOI:** 10.3390/tropicalmed4030100

**Published:** 2019-07-04

**Authors:** Shaun P. Coutts, Colleen L. Lau, Emma J. Field, Michael J. Loftus, Ee Laine Tay

**Affiliations:** 1 Health Protection Branch, Victorian Department of Health and Human Services, Melbourne, Victoria 3000, Australia; 2 Research School of Population Health, Australian National University, Canberra, Australian Capital Territory 2601, Australia; 3 Alfred Health, Melbourne, Victoria 3000, Australia

**Keywords:** Buruli ulcer, Australia, epidemiology, Mycobacterium ulcerans, skin ulcer, Tuberculosis and other mycobacteria

## Abstract

Uncertainty regarding transmission pathways and control measures makes prompt presentation and diagnosis for Buruli ulcer critical. To examine presentation and diagnosis delays in Victoria, Australia, we conducted a retrospective study of 703 cases notified between 2011 and 2017, classified as residing in an endemic (Mornington Peninsula; Bellarine Peninsula; South-east Bayside and Frankston) or non-endemic area. Overall median presentation delay was 30 days (IQR 14–60 days), with no significant change over the study period (*p* = 0.11). There were significant differences in median presentation delay between areas of residence (*p* = 0.02), but no significant change over the study period within any area. Overall median diagnosis delay was 10 days (IQR 0–40 days), with no significant change over the study period (*p* = 0.13). There were significant differences in median diagnosis delay between areas (*p* < 0.001), but a significant decrease over time only on the Mornington Peninsula (*p* < 0.001). On multivariable analysis, being aged <15 or >65 years; having non-ulcerative disease; and residing in the Bellarine Peninsula or South-East Bayside (compared to non-endemic areas) were significantly associated with shorter presentation delay. Residing in the Bellarine or Mornington Peninsula and being notified later in the study period were significantly associated with shorter diagnosis delay. To reduce presentation and diagnosis delays, awareness of Buruli ulcer must be raised with the public and medical professionals, particularly those based outside established endemic areas.

## 1. Introduction

**Figure 1:**
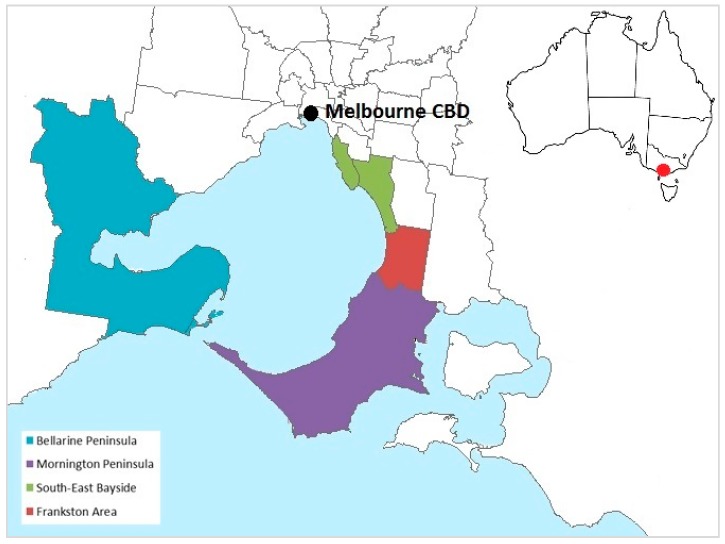
Geographic areas in the state of Victoria, Australia, classified as endemic for Buruli ulcer for the purposes of the study, based on local government area boundaries. Non-shaded areas and areas not pictured were considered non-endemic. Inset shows the location of Melbourne, Victoria, within Australia.

Buruli ulcer is a destructive bacterial infection of skin and soft tissue caused by the toxin-producing environmental pathogen *Mycobacterium ulcerans*, which is most prevalent in sub-Saharan Africa [1]. Buruli ulcer is also an escalating public health issue in the temperate Australian state of Victoria, with incidence increasing since 2012 to a record high of 340 cases in 2018 [2]. The majority of Victoria’s 6.4 million population reside in the metropolitan area of the state capital, Melbourne. Cases occur in residents and visitors to low-lying coastal areas considered endemic for the disease (Figure 1), many of which receive large numbers of visitors from non-endemic regions during summer. There is a seasonal pattern to disease onset—most infections become clinically apparent in autumn and winter, reflecting likely acquisition during summer or autumn based on an incubation period of up to nine months (median 4.8 months) [3,4]. Since 2012, incidence has declined in the long-established Bellarine Peninsula endemic area, but has increased rapidly on the Mornington Peninsula, and to a lesser extent in the South-East Bayside suburbs [3].

Uncertainties about the exact mode of transmission, environmental reservoirs and drivers of emergence have hampered the design and implementation of effective interventions to reduce disease transmission. However, basic preventative measures such as avoiding mosquito bites and skin abrasions have been promoted by health authorities [5].

Buruli ulcer may manifest as an ulcer, papule, subcutaneous nodule or raised plaque. If untreated, it can progress to significant ulceration, tissue loss and bone involvement, resulting in permanent disfigurement and long-term morbidity [1]. Less commonly, the disease may present as an oedematous lesion, often characterised by an intact dermis with cellulitis and low-grade fever. This form of the disease can be rapidly progressive and lead to extensive tissue loss. Oedematous lesions may be misdiagnosed as cellulitis, leading to delays in the diagnosis and treatment [6]. Combination oral antibiotic therapy (rifampicin with clarithromycin or a fluoroquinolone) for a minimum of eight weeks is the first-line treatment for uncomplicated Buruli ulcer in Victoria [7]. Successful antibiotic treatment is often followed by prolonged wound healing time; a recent study in Victoria described a median of 138 days (interquartile range (IQR) 91–175 days) [8].

Given the current lack of effective interventions to reduce transmission, prompt diagnosis and treatment remain paramount. Delays in seeking medical care, confirming diagnosis, and commencing treatment can contribute significantly to morbidity through extended treatment and healing times, long-term disfigurement, and increased treatment costs [6,9,10]. Since a 2007 study focused on cases on the Bellarine Peninsula between 1998 and 2006 [11], there has been little research on factors influencing presentation and diagnosis delays in Victoria. Using routine surveillance data, this study aimed to characterise presentation and diagnosis delays for Buruli ulcer cases notified to the Victorian Department of Health and Human Services (DHHS) between 2011 and 2017, and identify factors influencing these delays.

## 2. Materials and Methods

### 2.1. Study Population

Buruli ulcer is a notifiable condition in Victoria, with mandatory reporting by clinicians and laboratories to DHHS. The initial study population included all laboratory-confirmed cases in Victorian residents diagnosed in Victoria and notified to DHHS from 2011 to 2017. If case presentation or diagnosis delay could not be ascertained due to missing data, they were excluded from the final study population. 

### 2.2. Data Sources

Since 2011, Buruli ulcer case surveillance data were collected by the Victorian DHHS from medical practitioners using a standard surveillance form that includes demographic, clinical, treatment and risk history information. Data relevant to presentation and diagnosis delays are the date of first health care presentation, date of symptom onset, duration of symptoms before seeking care, and date on which Buruli ulcer was first clinically suspected. Many cases were initially diagnosed by a primary care doctor before referral to an infectious diseases specialist, meaning that the surveillance form may be completed by more than one medical practitioner. DHHS staff endeavour to contact all cases without a known link to a recognised endemic area by telephone for a detailed interview using a standard questionnaire. Data collected on enhanced surveillance forms and questionnaires were recorded in an electronic database.

### 2.3. Definitions

A confirmed case of Buruli ulcer required definitive laboratory evidence of infection, defined as the detection and specific identification of *Mycobacterium ulcerans* by culture on a specimen of tissue or a swab from a lesion (by the Mycobacterium Reference Laboratory at the Victorian Infectious Diseases Reference Laboratory) or the detection of the IS2404 insertion sequence by polymerase chain reaction.

Residential location was defined as the geographic area where the case was living at the time of notification. Geographic areas of residence were categorised into four areas (based on local government area boundaries) considered endemic for Buruli ulcer in Victoria—Mornington Peninsula, Bellarine Peninsula, South-east Bayside and Frankston Area. All other areas of Victoria were categorised non-endemic. 

Lesion severity was classified as per World Health Organization definitions [12]. Category I was defined as a single lesion <5 cm diameter; Category II a single lesion 5–15 cm diameter; Category III a single lesion >15 cm, multiple lesions, lesions at a critical site (e.g., the eye) or osteomyelitis. 

Presentation delay was defined as days from symptom onset to first presentation to a medical practitioner. Diagnosis delay was defined as days from presentation to a medical practitioner to first clinical suspicion of Buruli ulcer.

### 2.4. Data Analysis

De-identified data were extracted into Microsoft Excel and imported into STATA 15.1 (College Station, TX, USA) for analyses. 

Data were descriptively analysed to characterise the study population. Presentation and diagnosis delays were described using median, IQR and range. The significance of change over time in median presentation and diagnosis delays were assessed using Kruskal–Wallis tests.

Univariate Cox’s proportional hazards regression was used to identify significant associations between each dependent variable (presentation delay, diagnosis delay) and independent variables (age group, gender, year of notification, residential location at the time of notification, manifestation, lesion location, WHO lesion category). As the time-dependent variables represent a positive outcome (presentation or diagnosis), a hazard ratio of >1 indicates an association with shorter delay. Significance was assessed by the likelihood ratio test, with all independent variables with a *P*-value of <0.25 on univariate analysis considered for inclusion in a full multivariate model. A backward stepwise regression procedure (*p* ≤ 0.05) was performed to refine and select the final variables for the main effects model. Proportionality assumptions were tested using Schoenfeld and scaled Schoenfeld residuals. The fit of the final model was evaluated using Cox–Snell residuals. Any presentation or diagnosis delays recorded as zero days in the dataset were re-coded as 0.01 days for this analysis. 

### 2.5. Ethics

Human research ethics approval was granted by the Australian National University Human Research Ethics Committee on 17 July 2018 (2018/442). 

## 3. Results

### 3.1. Characteristics of the Study Population

Between 2011 and 2017, 877 confirmed cases of Buruli ulcer were notified to DHHS, and 703 (80%) cases were included in the study. Excluded were 174 cases for which presentation or diagnosis delay could not be ascertained from surveillance data. Significant differences were noted between the included and excluded cases for the clinical variables of lesion location, manifestation and WHO lesion category, likely because these data were collected primarily via surveillance forms which were not consistently completed by clinicians. Residential location differed between the included and excluded cases, reflecting more intensive public health follow-up of cases notified outside recognised endemic areas. The characteristics of the total, excluded and included cases are described in Table 1.

**Figure 2:**
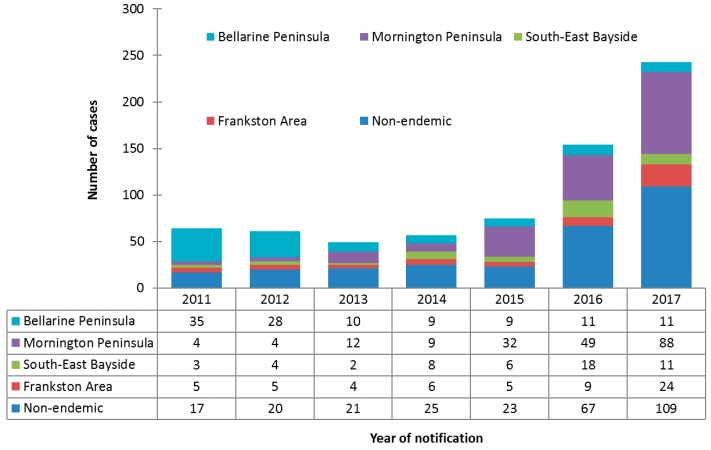
Counts and proportionate distributions of Buruli ulcer cases in the study population notified to the Department of Health and Human Services from 2011 to 2017, by area of residence (*n* = 703).

Incidence increased over the study period in all areas except the Bellarine Peninsula, where incidence decreased from 35 cases in 2011 to 11 cases in 2017. The greatest increase in incidence was observed for cases on the Mornington Peninsula, from four cases in 2011 to 88 cases in 2017. Cases residing in non-endemic areas also increased significantly over the study period, from 17 cases in 2011 to 109 cases in 2017. The number and proportionate distribution of cases over the study period by area of residence is summarized in Figure 2.

### 3.2. Presentation and Diagnosis Delays

#### 3.2.1. Presentation Delay

**Figure 3:**
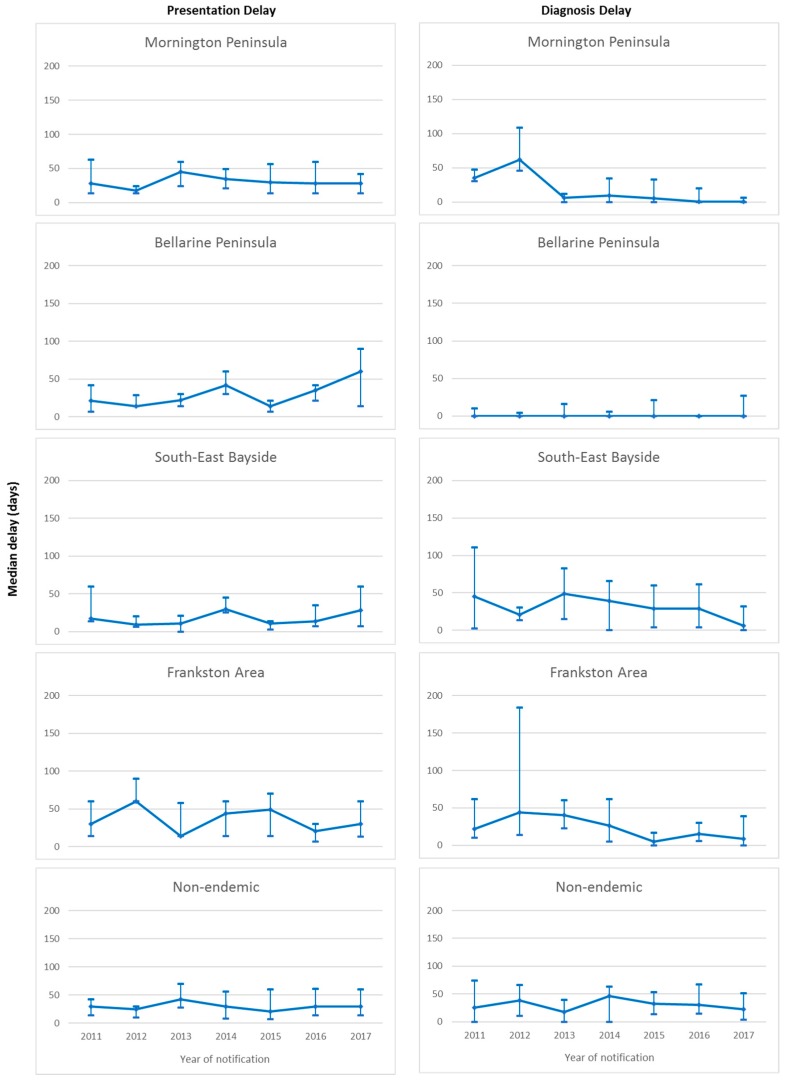
Median and interquartile ranges for presentation and diagnosis delays (days) of Buruli ulcer cases in the study population notified to the Victorian Department of Health and Human Services from 2011 to 2017, by area of residence.

Overall median presentation delay was 30 days (IQR 14–60 days), with no significant change over the study period (*p* = 0.11). Significant differences in median presentation delay between areas of residence were observed over the study period (*p* = 0.02), with shortest delay in South-east Bayside (19 days, IQR 7–35.5 days), followed by the Bellarine Peninsula (21 days, IQR 14–42 days), Mornington Peninsula (29.5 days, IQR 14–56 days), and Frankston and non-endemic areas (both 30 days, IQR 14–60 days). No significant change in median presentation delay was observed within any of the areas of residence over the study period (Figure 3). 

#### 3.2.2. Diagnosis Delay

Overall median diagnosis delay was 10 days (IQR 0–40 days), with no significant change observed over the study period (*p* = 0.13). Significant differences in median diagnosis delay between areas of residence were observed (*p* < 0.001). Median diagnosis delay was shortest on the Bellarine Peninsula (0 days, IQR 0–7 days) and the Mornington Peninsula (0 days, IQR 0–19 days), followed by Frankston (16 days, IQR 3–45 days), South-east Bayside (20.5 days, IQR 1–61 days) and non-endemic areas (29 days, IQR 7–56 days). Significant decrease in median diagnosis delay over the study period was observed only for the Mornington Peninsula (*p* < 0.001), however the median remained at zero days for all years of the study period on the Bellarine Peninsula. Non-significant decreases were observed in South-East Bayside and Frankston. No decrease was observed for the non-endemic areas. Median diagnosis delay over the study period by area of residence is illustrated in Figure 3.

### 3.3. Factors Influencing Presentation and Diagnostic Delays

#### 3.3.1. Presentation Delay

Table 2 provides a summary of associations between independent variables and presentation delay on univariate and multivariate Cox regression analysis. In the final multivariate model, being aged <15 years or >65 years, having non-ulcerative disease, and residing in the Bellarine Peninsula or South-East Bayside compared to a non-endemic area remained significantly associated with shorter presentation delay. 

#### 3.3.2. Diagnosis Delay

Table 3 provides a summary of associations between independent variables and diagnosis delay on univariate and multivariate Cox regression analysis. In the final multivariate model, residing in the Bellarine Peninsula or Mornington Peninsula compared to a non-endemic area and being notified later in the study period remained significantly associated with shorter diagnosis delay.

## 4. Discussion

This study found that despite a significant difference in median presentation delay between areas of residence, presentation delay had not significantly decreased in Victoria as a whole, or in any specific area during the period 2011–2017. There was a significant association between shorter presentation delays and residence in South-East Bayside or Bellarine Peninsula. This is consistent with a recent study by Loftus et al. (using a similar surveillance dataset covering 2011–2016), which identified shortest presentation delays for cases with likely exposure to the disease on the Bellarine Peninsula compared to the rest of Victoria, however the difference was not considered significant [3]. Significant association between cases aged <15 years or >65 years and shorter presentation delays is also consistent with previous findings [3]. Significant association between shorter presentation delays and having non-ulcerative disease has been previously identified in a study in Benin [13]. 

There was no significant decrease in median diagnosis delay in Victoria as a whole, however a significant decrease was observed on the Mornington Peninsula even as case numbers increased year on year, reaching a zero-day median in 2016. There was a significant association between shorter diagnosis delays and residence in Bellarine or Mornington Peninsula, and with diagnosis later in the study period. These findings are consistent with previous studies in Victoria, which identified shortest diagnosis delays in established endemic areas [3,11,14]. A recent study using a similar surveillance dataset found that most cases in non-endemic areas had a likely exposure to one or more of the endemic areas during their plausible acquisition period [3].

The zero-day median diagnosis delay on the Bellarine Peninsula throughout the study period is likely reflective of its longer history as an endemic area and higher clinical index of suspicion among local medical practitioners. Likewise, the significant decrease on the Mornington Peninsula is likely related to growing medical practitioner and resident familiarity with the disease over the study period as case numbers increased locally and the disease received additional attention from public health authorities, the medical community and the media. The results of the multivariate analysis support this hypothesis, with residence in the two areas that have previously or are currently experiencing intense endemic transmission being associated with significantly shorter diagnosis delay. DHHS has made significant efforts to raise awareness of the disease as it has emerged in these areas, including advisory notices from the state’s Chief Health Officer and an online continuing professional development module for primary care doctors [5,15].

The association between shorter presentation delays and cases being aged <15 years or >65 years may be due to an increased caution about health issues in children and older people. A previous Victorian study found that children and older people are at greater risk of severe Buruli ulcer disease [2]. The association between shorter presentation delays and non-ulcerative disease may be because these symptoms (i.e., oedema, cellulitis and plaques) are less likely to be dismissed as insect bites, are often rapidly progressive and can involve systemic symptoms like fever. 

This study utilized a large, robust surveillance dataset and included 80% of cases notified in Victoria over the study period. Limitations included the retrospective nature of surveillance data, and exclusion of cases due to missing data, and the fact that the majority of cases resided in endemic areas, which may have biased the data. We were also unable to include other factors that may impact on presentation and diagnosis delays such as medical co-morbidities, occupation, social connectivity, socioeconomic status, educational level or geographic distance to health care facilities as these are not collected as part of routine surveillance. Place of residence was recorded only at the time of notification and may be different to when the disease was acquired or diagnosed, due to the potentially extended incubation period and delays in presentation and diagnosis.

On a global scale, delays in presentation and diagnosis for Buruli ulcer in Victoria are relatively short—a study of 82 Nigerian patients in Benin identified a median delay from symptom onset to diagnosis of 29 weeks (IQR 12–234) [16]. Lengthy delays have also been reported in Cameroon (median 12 weeks, IQR 3–30, *n* = 105) and Nigeria (median 12 weeks, IQR 6–50, *n* = 145) [17,18]. It is important to recognise the differing drivers for delays in each context—whilst geographical and economic inaccessibility to health care and an initial preference for traditional healing methods have been identified in Africa, [16,19] these are highly unlikely to be important factors in Victoria.

To further reduce delays in diagnosis, public health authorities must continue to find ways to engage with and effectively raise clinician awareness of Buruli ulcer, particularly those based outside the recognised endemic areas. Clinicians may be unfamiliar with or have a low level of clinical suspicion if patients acquired Buruli ulcer many months earlier while visiting an endemic area. This contention is supported by the relatively lengthy diagnosis delays observed for cases residing in non-endemic areas compared to those in recognised endemic areas, and the lack of an observable decrease in median diagnosis delay over the study period. 

The absence of a decrease in presentation delay over the study period suggests that a review of public health communications and community engagement (including the importance of early presentation, early diagnosis and preventative measures) may be warranted. In a study of 85 patients in the Bellarine Peninsula, seeing media related to Buruli ulcer was a significant factor in seeking diagnosis for nearly one quarter of cases [11]. A review of communications and community engagement could gauge the effectiveness of current strategies for raising awareness among local communities and visitors to endemic areas and identify areas for improvement. 

As Buruli ulcer incidence continues to increase in Victoria, effective interventions to reduce transmission are limited due to continuing uncertainty regarding the modes of transmission. Effective risk communication and awareness remain critical to reducing the disease burden.

## Figures and Tables

**Table 1:** Characteristics of Buruli ulcer cases (total, included and excluded for analysis) notified to the Victorian Department of Health and Human Services from 2011 to 2017.

	Total Notifications		Included for Analysis		Excluded for Analysis		*P*-Value
	no.	%	no.	%	no.	%	
**Sex**							0.15
Female	391	44.6	305	43.4	86	49.4	
Male	486	55.4	398	56.6	88	50.6	
**Age group**							0.95
<15 years	96	11.0	77	11.0	19	10.9	
15–65 years	495	56.4	395	56.2	100	57.5	
>65 years	286	32.6	231	32.9	55	31.6	
**Residential location**							<0.001
Bellarine Peninsula	184	21.0	113	16.1	71	40.8	
Mornington Peninsula	245	27.9	198	28.2	47	27.0	
South-East Bayside	63	7.2	52	7.4	11	6.3	
Frankston Area	75	8.6	58	8.3	17	9.8	
Non-endemic	310	35.4	282	40.1	28	16.1	
**Lesion location**							<0.001
Upper Limb	199	22.7	167	23.8	32	18.4	
Lower Limb	515	58.7	425	60.5	90	51.7	
Other *	104	11.9	91	12.9	13	7.5	
Unknown	59	6.7	20	2.8	39	22.4	
**Manifestation**							<0.001
Ulcer	644	73.4	549	78.1	95	54.6	
Non-ulcer †	154	17.6	138	19.6	16	9.2	
Unknown	79	9.0	16	2.3	63	36.2	
**WHO lesion category**							<0.001
I	619	70.6	539	76.7	80	46.0	
II	110	12.5	91	12.9	29	10.9	
III	60	6.8	55	7.8	5	2.9	
Unknown	88	10.0	18	2.6	70	40.2	

* includes lesions on areas of the body other than limbs, and lesions in multiple locations (including limbs). † includes all non-ulcerative Buruli ulcer manifestations.

**Table 2:** Associations between independent variables and presentation delay on univariate and multivariate Cox’s regression analysis.

	Observations	Median Delay (days)	Univariate			Multivariate		
			Crude HR	95% CI	*P*-Value	Adjusted HR	95% CI	*P*-Value
**Sex**								
Female	305	28	1.06	0.91–1.23	0.47			
Male	398	30	Reference					
**Age group**								
<15 years	77	**21**	**1.31**	**1.02–1.67**	**0.03**	**1.35**	**1.04–1.73**	**0.02**
15–65 years	395	30	Reference			Reference		
>65 years	231	**23**	**1.29**	**1.09–1.52**	**0.002**	**1.31**	**1.10–1.55**	**0.002**
**Residential location**								
Bellarine Peninsula	113	**21**	**1.20**	**0.97–1.50**	**0.10**	**1.26**	**1.01–1.58**	**0.04**
Mornington Peninsula	198	29.5	1.07	0.89–1.28	0.48	1.06	0.88–1.28	0.53
South-East Bayside	52	**19**	**1.40**	**1.04–1.88**	**0.03**	**1.44**	**1.06–1.95**	**0.02**
Frankston area	58	30	0.88	0.66–1.17	0.38	0.87	0.65–1.17	0.36
Other (non-endemic)	282	30	Reference			Reference		
**Lesion location**								
Upper limb	167	30	Reference					
Lower limb	425	30	1.04	0.87–1.24	0.69			
Other *	91	21	1.16	0.89–1.49	0.27			
**Manifestation**								
Ulcer	549	30	Reference			Reference		
Non-ulcer †	138	**21**	**1.28**	**1.06–1.54**	**0.01**	**1.27**	**1.05–1.53**	**0.02**
**Year of notification**								
Increasing from 2011	703	**-**	**0.97**	**0.93–1.00**	**0.06**			
**WHO lesion category**								
I	539	30	1.07	0.85–1.33	0.57			
II	91	28	Reference					
III	55	**28**	**1.30**	**0.93–1.81**	**0.13**			

* includes lesions on areas of the body other than limbs, and lesions in multiple locations (including limbs). † includes all non-ulcerative Buruli ulcer manifestations. HR = hazard rate CI = confidence interval. **Bold type** indicates significance for inclusion in the full and main effects models.

**Table 3:** Associations between independent variables and diagnosis delay on univariate and multivariate Cox’s regression analysis.

	Observations	Median Delay (days)	Univariate			Multivariate		
			Crude HR	95% CI	*P*-Value	Adjusted HR	95% CI	*P*-Value
**Sex**								
Female	**305**	**14**	**0.89**	**0.77–1.04**	**0.14**			
Male	398	7	Reference					
**Age group**								
<15 years	**77**	**19**	**0.84**	**0.66–1.08**	**0.17**			
15–65 years	395	13	Reference					
>65 years	231	7	1.08	0.92–1.28	0.33			
**Residential location**								
Bellarine Peninsula	113	**0**	**2.63**	**2.09–3.30**	**<0.001**	**3.18**	**2.47–4.09**	**<0.001**
Mornington Peninsula	198	**0**	**1.97**	**1.63–2.37**	**<0.001**	**1.94**	**1.60–2.35**	**<0.001**
South-East Bayside	52	20.5	1.04	0.77–1.39	0.82	1.07	0.79–1.44	0.67
Frankston area	58	16	1.17	0.88–1.55	0.28	1.21	0.91–1.61	0.19
Other (non-endemic)	282	29	Reference			Reference		
**Lesion location**								
Upper limb	167	12	Reference					
Lower limb	425	13	1.02	0.85–1.22	0.823			
Other *	91	6	1.14	0.88–1.47	0.32			
**Manifestation**								
Ulcer	549	10	Reference					
Non-ulcer †	138	13.5	0.95	0.79–1.15	0.60			
**Year of notification**								
Increasing from 2011	703	**-**	**1.03**	**0.99–1.06**	**0.19**	**1.09**	**1.04–1.13**	**<0.001**
**WHO lesion category**								
**I**	**539**	**9**	**1.26**	**1.01–1.58**	**0.04**			
II	91	19	Reference					
III	55	31	0.87	0.62–1.22	0.41			

* includes lesions on areas of the body other than limbs, and lesions in multiple locations (including limbs). † includes all non-ulcerative Buruli ulcer manifestations. HR = hazard rate CI = confidence interval. **Bold type** indicates significance for inclusion in the full and main effects models.
